# Flecainide Toxicity Secondary to Accidental Overdose: A Pediatric Case Report of Two Brothers

**DOI:** 10.1155/2021/6633859

**Published:** 2021-05-13

**Authors:** Sarah E. Gardner Yelton, James B. Leonard, Caridad M. de la Uz, Rajeev S. Wadia, Sean S. Barnes

**Affiliations:** ^1^Department of Anesthesia and Critical Care Medicine, Charlotte R. Bloomberg Children's Center, Johns Hopkins University School of Medicine, Baltimore, Maryland, USA; ^2^Maryland Poison Center, Baltimore, Maryland, USA; ^3^Department of Pediatrics, Division of Pediatric Cardiology, Charlotte R. Bloomberg Children's Center, Johns Hopkins University School of Medicine, Baltimore, Maryland, USA

## Abstract

Flecainide is a class 1C antiarrhythmic with a narrow therapeutic window and thereby a high-risk medication for causing acute toxicity. Dysrhythmias secondary to flecainide ingestion are often refractory to antiarrhythmics and cardioversion, and patients commonly require extracorporeal support. We review the successful resuscitation of two brothers aged 2 and 4 who presented two years apart with unstable wide-complex tachyarrhythmia suspicious for severe flecainide toxicity. Each patient received sodium bicarbonate and 20% intravenous lipid emulsion with a full recovery. While extracorporeal support is often required following flecainide ingestion, we present two cases where it was avoided due to aggressive multimodal management with sodium bicarbonate, electrolyte repletion, and 20% intravenous lipid emulsion. In addition, avoidance of agitation-induced tachycardia may be beneficial.

## 1. Introduction

Flecainide is a class 1C antiarrhythmic commonly used in pediatrics to treat refractory supraventricular arrhythmias [1]. It has a narrow therapeutic index with goal trough levels ranging from 0.2 *μ*g/mL to 1 *μ*g/mL but toxic levels as low as 0.7 *μ*g/mL and a mortality rate of approximately 22.5% [1–3]. Maximum recommended pediatric daily dosing is 200 mg/m^2^/day [1]. Flecainide blocks fast sodium channels to slow cardiac conduction, therefore widening the QRS complex and prolonging both the QT and PR intervals [1]. Signs of serious intoxication include altered mental status, seizures, hypotension, ventricular tachydysrhythmia, severe bradycardia, and AV block [1]. Patients with structural heart disease are particularly vulnerable to arrhythmias [4]. Although there are some reports of successful cardioversion with usual therapies (i.e., amiodarone, lidocaine, and defibrillation), flecainide-induced dysrhythmias are often refractory to these treatments [5, 6]. Alternative therapies include sodium bicarbonate, 20% intravenous lipid emulsion (ILE), and progression to extracorporeal support. However, there are no randomized controlled trials to support these treatments, and neither sodium bicarbonate nor ILE is approved by the Food and Drug Administration for the indication of flecainide overdose [1]. We describe the clinical presentation of two children with flecainide toxicity and review their life-saving resuscitation.

## 2. Case Presentation

Informed parental consent was obtained to report the following cases.

### 2.1. Case 1

**Figure 1:**
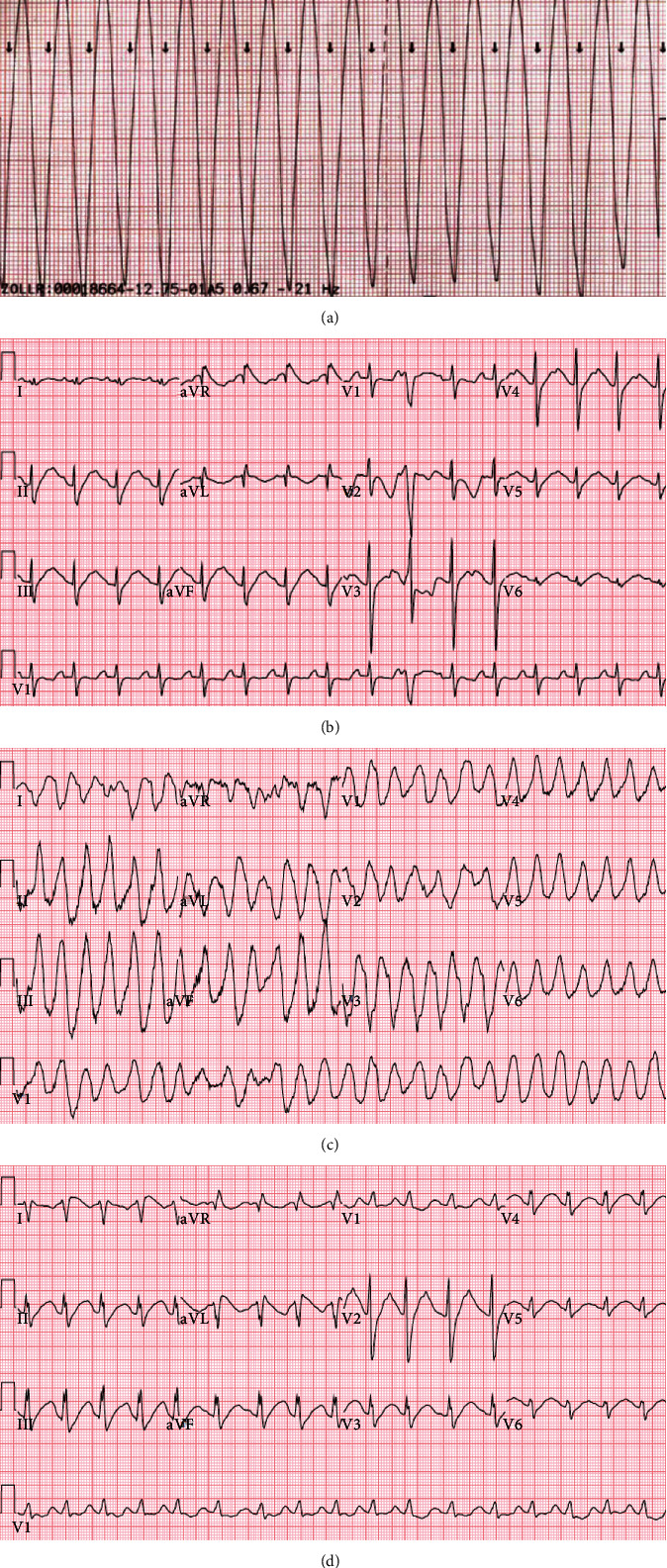
(a) Child 1 rhythm strip on presentation. (b) Child 1 electrocardiogram (EKG) on pediatric intensive care unit admission. (c) Child 2 EKG in emergency department. (d) Child 2 EKG following magnesium administration.

Child 1 is a 4-year-old male with permanent junctional reciprocating tachycardia (PJRT) on flecainide maintenance therapy (30 mg every 8 hours). He presented to the emergency department three hours after an unwitnessed ingestion of approximately 2 g of flecainide with alternating bradycardia and wide complex tachyarrhythmia (Figure 1(a)), altered mental status, hypotension, and poor perfusion. Presenting heart rate varied from 60 beats per minute (BPM) to 160 BPM, blood pressure was 50/30 mmHg with weak central pulses. Although the patient was breathing spontaneously with oxygen saturations of 100% on a nonrebreather mask, he was responsive only to pain. Initial pH was 7.13 with a pCO_2_ of 71 mmHg and a serum bicarbonate level of 23 mmol/L. Flecainide trough level on arrival was 5.6 *μ*g/mL (therapeutic range 0.2 *μ*g/mL to 1 *μ*g/mL). Synchronized cardioversion was performed three times (0.5 J/kg, 1 J/kg, 2 J/kg), and 1-2 mEq/kg 8.4% sodium bicarbonate boluses were given three times, resulting in conversion to sinus rhythm with widened QRS (Figure 1(b)). The patient was transferred to the pediatric intensive care unit (PICU) with consultation from the local poison center.

On admission to the PICU, the patient's heart rate was 160 BPM with no other changes to his physical exam or vital signs. He was intubated and mechanically ventilated with extracorporeal support and a pediatric cardiac electrophysiology consultant immediately available. The patient's serum potassium was 2.5 mEq/L; ionized calcium was 0.9 mmol/L with otherwise normal electrolytes. Sodium bicarbonate boluses were given in 1-2 mEq/kg increments for QRS > 100 ms to maintain a goal pH of 7.5. He was initiated on a sodium bicarbonate infusion at a rate of 1 mEq/kg/hr which continued until the following morning. Serum calcium and potassium were replaced and magnesium given for treatment of the wide complex arrhythmia. Due to continued dysrhythmia and hemodynamic instability despite the above treatments, the child received 1.5 mL/kg 20% ILE bolus followed by a 0.25 mL/kg/min infusion. Following, the child had successful conversion to a sinus rhythm at a rate of 80 BPM with a wide QRS, blood pressure of 90/60 mmHg, and improved perfusion and mental status. The ILE infusion was weaned and completely discontinued after two hours. The child continued to have intermittent runs of pleomorphic ventricular tachycardia with agitation while mechanically ventilated. These episodes of agitation-induced tachycardia were successfully treated with an appropriate sedative dose. Following hemodynamic stability, the child was extubated after 36 hours, at which time the QRS had completely normalized. The patient's serum flecainide level had decreased to 0.3 *μ*g/mL, and his home dose of flecainide restarted. He was discharged home on the same maintenance dose of 30 mg every 8 hours.

### 2.2. Case 2

Child 2, a 2-year-old male with history of fetal supraventricular tachycardia (resolved), presented to the emergency department with seizure-like activity, altered mental status, bradycardia, and hypotension. Although serum flecainide concentrations were not obtained, the patient's mother reported finding the child near his brother's (child 1) empty flecainide bottle, estimating approximately a 400 mg ingestion 45 minutes prior to presentation. This occurred two years following his brother's hospitalization. On arrival to the hospital, his heart rate was 70 BPM, blood pressure 70/30 mmHg with faint central pulses, and capillary refill time of 4 seconds. He was breathing spontaneously with an oxygen saturation of 100% on a nonrebreather mask. He was responsive only to pain. Presenting pH was 7.2 with a pCO_2_ of 63 mmHg and a serum bicarbonate level of 24 mmol/L. After receiving 1 mg/kg lidocaine bolus with no effect, he was given three 0.5 mEq/kg sodium bicarbonate boluses resulting in wide complex tachycardia (Figure 1(c)). He was then transferred to the PICU. The local poison center was consulted.

On admission to the PICU, the patient's heart rate was 150 BPM with a blood pressure of 64/40 mmHg but otherwise no change in his physical exam or vital signs. The child was intubated and mechanically ventilated with extracorporeal support and a pediatric cardiac electrophysiologist immediately available, as above. Sodium bicarbonate boluses were given in 1-2 mEq/kg increments for QRS > 100 ms to maintain a goal pH of 7.5. Serum potassium was 3.3 mEq/L, and ionized calcium was 0.91 mmol/L, both of which were repleted. All other electrolytes were normal. A 50 mg/kg intravenous dose of magnesium sulfate was given for the wide complex tachyarrhythmia with brief conversion to sinus rhythm (Figure 1(d)).

An isoproterenol infusion was initiated in an attempt to suppress recurrent ventricular tachycardia at 0.1 mcg/kg/min, resulting in persistent widening of the QRS, so the infusion was stopped. He also received 1 mEq/kg 3% hypertonic saline. Due to continued dysrhythmia and hemodynamic instability despite the above interventions, the child received 1.5 mL/kg 20% ILE bolus followed by a 0.25 mL/kg/min infusion. He required a second bolus of 1.5 mL/kg 20% ILE due to persistent hypotension, followed by an increase in the infusion rate to 0.5 mL/kg/min. With the above interventions, the child had successful conversion to a sinus rhythm at a rate of 90 BPM with a wide QRS, blood pressure of 100/50 mmHg, strong pulses, and capillary refill time of 2 seconds. The ILE infusion was weaned and completely discontinued after two hours. Similar to his brother, the patient continued to have intermittent runs of pleomorphic ventricular tachycardia with agitation while mechanically ventilated, which resolved with appropriate sedative dosing. Following hemodynamic stability, child 2 was extubated after 24 hours of mechanical ventilation with no remaining sedative requirement. Complete normalization of the QRS took approximately 36 hours. Child protective services was notified in both cases, and a safety plan for discharge was determined.

## 3. Discussion

Management of flecainide toxicity can be challenging. We described the presentation and successful treatment of two children, which precluded the need for extracorporeal support. In cases of severe intoxication and hemodynamic instability, we recommend an integrated approach with sodium bicarbonate, normalization of electrolytes, ILE, avoidance of agitation, mechanical ventilatory support, and immediate availability of extracorporeal support.

Sodium bicarbonate can be used as monotherapy and is commonly given as the first line treatment for QRS > 100 ms in increments of 1-2 mEq/kg [1, 7–9]. It increases the availability of extracellular sodium to compete with flecainide for binding sodium channels and raises the serum pH to increase the electrochemical gradient across cell membranes [10]. In animal models, sodium bicarbonate decreases QRS prolongation caused by flecainide and improves survival when compared with normal saline [11, 12]. Although child 1 received a bicarbonate infusion, administering serial bolus doses is considered more effective. Alkalization of the urine following bicarbonate administration may delay clearance of flecainide, so adjunctive therapies have been proposed [1]. Limited data supports the use of 3% hypertonic saline as an adjunct to sodium bicarbonate administration [13]. Additionally, isoproterenol has been used to reverse toxic effects of flecainide by increasing inward sodium current [14].

The rationale for using intravenous lipid emulsion (ILE) in flecainide toxicity is extrapolated from its use in the treatment for regional anesthetic drug toxicity [15, 16]. Similar to flecainide, regional anesthetic drugs (i.e., bupivacaine and ropivacaine) are also lipophilic and block sodium channels. ILE creates a “lipid sink” that removes any lipophilic drug from the intravascular space by incorporating it into the fat globules of the lipid, thus sequestering it from the remainder of the body [15, 17, 18]. Additionally, it increases inotropy [19]. In a systematic review, Jamaty et al. recommend ILE for local anesthetic toxicity in the setting of neurologic or cardiovascular deterioration, in addition to hemodynamic instability from intoxication from other fat-soluble drugs, after other supportive measures and antidotes have been unsuccessful [20]. In the case of flecainide ingestion, collaborative guidelines from several toxicology societies give ILE use a neutral recommendation if cardiac arrest or potential life-threatening ingestion is present and recommend against its use for non-life-threatening ingestions [21, 22]. Most commonly cited dosing is reported in Table 1. There have been reports of fat emboli, pancreatitis, and acute respiratory distress syndrome with administration of large doses of ILE and decreasing the infusion rate followed by ILE discontinuation as soon as hemodynamic stability is achieved is suggested [20–24]. The most common side effect, which occurred in our case, is lipemia, which can interfere with serum analysis and complicates laboratory interpretation [20].

Rhythm response to normalization of electrolytes has been reported, and as per pediatric advanced life support recommendations, magnesium should be administered for polymorphic ventricular tachycardia [1, 25, 26]. Extracorporeal support is commonly required for unstable refractory arrhythmias to support hemodynamics until drug clearance can occur [27, 28]. Flecainide clearance can be prolonged as the average half-life is 20 hours, and failure to provide hemodynamic support can result in death or permanent neurologic injury. The volume of distribution of flecainide precludes the use of dialysis and hemoperfusion for removal and is therefore not recommended [29].

Both children had progressive widening of the QRS complex with tachycardia in the setting of agitation during the first 12 hours postingestion. This association of tachycardia and QRS prolongation has been described in both human and animal models with maintenance flecainide doses, even in the absence of toxicity [30–32]. At a higher heart rate, more sodium channels are normally open, and since flecainide preferentially blocks open channels, its effects may be exacerbated [31]. To our knowledge, no other case reports describe induction of wide-complex tachycardia with agitation. In both children, this arrhythmia was successfully reversed with administration of sedation.

## 4. Conclusions

Flecainide ingestions can cause wide complex tachyarrhythmias and hemodynamic collapse refractory to typical management, often requiring extracorporeal support. While we cannot conclude with certainty if one treatment was solely responsible for the improvement observed in our patients, we do provide validation for a multimodal treatment approach to flecainide toxicity, preventing the need for extracorporeal support. Flecainide toxicity should be treated with sodium bicarbonate boluses; electrolyte repletion, with particular attention to magnesium: intravenous lipid emulsion; judicious administration of sedatives to avoid tachycardia from agitation; and if needed extracorporeal support.

## Figures and Tables

**Table 1:** 20% intravenous lipid emulsion dosing recommendations [20–24].

Initial dosing	Infusion	Refractory	Maximum dosing
1.5 mL/kg 20% ILE as IV bolus over 2-3 minutes	0.25 mL/kg/min for 60 min	If continued instability after 3-5 minutes of infusion, repeat initial bolus dose and increase infusion to 0.5 mL/kg/min. Can give additional bolus doses for clinical deterioration.	Continue infusion for 10 minutes after circulatory stability is achieved. 10-12 mL/kg cumulative dosing within first 30 minutes of administration.
